# High Relative Abundance of *Lactobacillus reuteri* and Fructose Intake are Associated with Adiposity and Cardiometabolic Risk Factors in Children from Mexico City

**DOI:** 10.3390/nu11061207

**Published:** 2019-05-28

**Authors:** Eira E. Huerta-Ávila, Ivonne Ramírez-Silva, Luisa E. Torres-Sánchez, Cinthya E. Díaz-Benítez, Yaneth C. Orbe-Orihuela, Alfredo Lagunas-Martínez, Marcia Galván-Portillo, Mario Flores, Miguel Cruz, Ana I. Burguete-García

**Affiliations:** 1 Centro de Investigación sobre Enfermedades Infecciosas, Instituto Nacional de Salud Pública, Cuernavaca, Morelos 62100, México; lneeha920609@gmail.com (E.E.H.-Á.); cediaz@insp.mx (C.E.D.-B.); jcitla_oro@hotmail.com (Y.C.O.-O.); alagunas@insp.mx (A.L.-M.); 2 Centro de Investigación sobre Nutrición y Salud, Instituto Nacional de Salud Pública, Cuernavaca, Morelos 62100, México; ciramir@insp.mx (I.R.-S.); mario.flores@insp.mx (M.F.); 3 Centro de Investigación Salud Poblacional, Instituto Nacional de Salud Pública, Cuernavaca, Morelos 62100, México; mgalvan@insp.mx (L.E.T.-S.); ltorress@insp.mx (M.G.-P.); 4 Unidad de Investigación Médica en Bioquímica, Centro Médico Nacional Siglo XXI, Instituto Mexicano del Seguro Social, Juárez, Ciudad de México, CDMX 06600, México; mcruzl@yahoo.com

**Keywords:** adiposity, cardiometabolic markers, children, cholesterol, fructose, *Lactobacillus reuteri*

## Abstract

In Mexico, 3 of 10 children are overweight. Fructose intake and relative abundance (RA) of *Lactobacillus reuteri* (*L. reuteri*) in the intestinal microbiota are associated with obesity and diabetes in adults, but studies in children are limited. This study evaluates the association between fructose intake and *L. reuteri* RA with adiposity and cardiometabolic risk markers in Mexican children dietary information, microbiota profiles, adiposity indicators (Body Mass Index, BMI and Waste Circumference, WC), and cardiometabolic markers were analyzed in 1087 children aged 6–12 years. Linear regression and path analysis models were used. High-tertile fructose intake and *L. reuteri* RA were positively associated with BMI (β_Tertil 3 vs. Tertil 1_ *=* 0.24 (95% CI, 0.04; 0.44) and β_T3 vs. T1_ = 0.52 (95% CI, 0.32; 0.72)) and WC (β_T3 vs. T1_ = 2.40 (95% CI, 0.93; 3.83) and β_T3 vs. T1_ = 3.40 (95% CI, 1.95; 4.90)), respectively. Also, these factors mediated by adiposity were positively correlated with high triglycerides and insulin concentrations and HOMA-IR (*p* ≤ 0.03) and negatively associated with HDL-C concentration (*p* < 0.01). High-tertile fructose intake and *L. reuteri* RA were directly associated with adiposity and indirectly associated though adiposity with metabolic disorders in children. In conclusion, fructose intake and *L. reuteri* RA were directly associated with adiposity and indirectly associated with metabolic disorders in children, mediated by adiposity.

## 1. Introduction

Worldwide, overweightness (OW) and obesity (OB) affect 340 million children and adolescents aged 5–19 [1]. Mexico has the fourth highest prevalence of OB in children according to the Organization for Economic Cooperation and Development [2]. OB results from a positive energy balance (energy intake exceeds expenditure), and genetic and environmental factors, like diet and microbiota, play important roles in modulating this balance. Nowadays, fructose, a component from diet, is added to a great proportion of industrialized food and beverages [3,4]. Systematic reviews of clinical assays in adults have shown that an excessive intake of fructose can increase triglyceride levels and corporal weight [5,6,7]. Sweetened soda intake has been positively associated with body weight gain, particularly in children [8,9,10]. Furthermore, high-tertile fructose intake has been associated with insulin resistance and type 2 diabetes [11]. On the other hand, differences in gut microbiota composition determine an individual’s capacity to obtain energy from food, contributing to predisposition to metabolic disorders such as OB and diabetes [12]. Gut microbiota composition is associated with these diseases due to its role in insulin signaling, inflammation, and fat storage modulation [13]. The microbiota regulates the lipid metabolism of the host by secondary bile acids and the metabolism of glucose through the farnesoid X and G-protein-coupled receptors [14]. Recently, some authors have reported that a relative abundance (RA) of *L. reuteri* from gut microbiota is positively correlated with corporal weight gain in adults [15,16]. Moreover, some studies have documented that *L. reuteri* may take advantage of fructose molecules by obtaining energy generated from electron exchange in order to increase its growth rate [17,18,19]. This mechanism contributes to blood absorption of high amounts of fructose, increasing the synthesis of intermediary molecules to produce triglycerides [20,21,22]. Although *L. reuteri* cannot catabolize fructose due to the lack of enzymes capable of degrading monosaccharides, intestinal microbiota contributes to the assimilation and adipose storage of ingested calories, helping their own proliferation. So far, the associations between fructose intake or *L. reuteri* RA with obesity have been studied separately in adults or animals but not in children. The relationships of these associations with cardiometabolic markers have also not been reported. Therefore, this study aimed to evaluate the associations between fructose intake and *L. reuteri* RA with adiposity. Furthermore, we analyzed whether fructose intake and *L. reuteri* RA are associated with cardiometabolic markers in children and whether adiposity mediates these relationships.

## 2. Materials and Methods

### 2.1. Study Design and Participants

We analyzed dietary information, microbiota profiles, and OW and OB statuses of children aged 6 to 12 years who participated in a cross-sectional study between June 2011 and July 2013. The study sample was selected from four regions of Mexico City (north, south, east, and west) through a non-probability simple sampling method. A more detailed description of the methodology used in the study is presented in another manuscript [23].

The selected participants were children with no history of diabetes, hypertension, or cardiovascular disease and with no physical limitation to the performance of the anthropometric measurements. We excluded children with an infectious disease or gastrointestinal disorder and children who had been taking antibiotics for two months prior to the study at the time of the interview.

**Figure 1:**
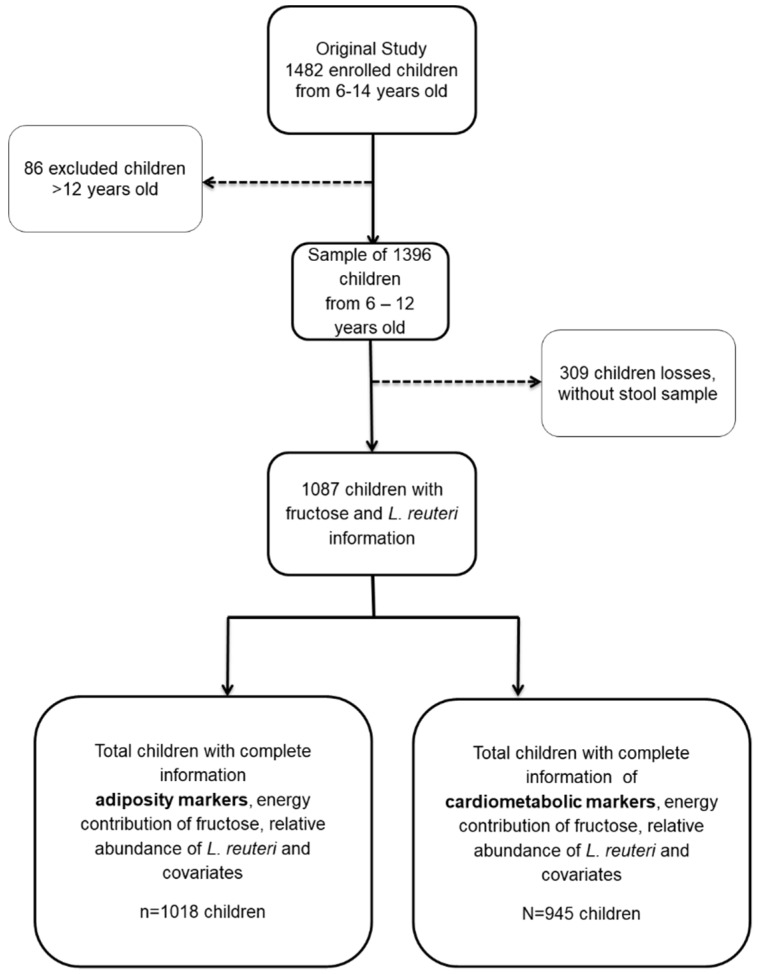
Children from México City study sample.

All subjects gave their informed consent and assent for inclusion prior to participation in the study (Figure 1). The study was conducted in accordance with the Declaration of Helsinki, and the protocol was approved by Ethics Committee of the National Institute of Public Health of Mexico (INSP) CI 1129 and the Social Security Mexican Institute (IMSS) 006-785-072.

### 2.2. Outcome Variables

#### 2.2.1. Adiposity

Trained personnel, previously standardized according to the Habicht method [24], carried out the anthropometric measurements. Scales with a precision of 0.1 kg, as well as a stadiometer and metric tape, both with precision of 1 mm (Seca GmbH. & Co., Hamburg, Germany), were used.

The weight (kg), height (cm), waist circumference (WC; cm), and hip circumference (cm) of participants were measured without shoes on and with the least amount of clothing possible, following the Lohman procedure [25]. Adiposity was evaluated by body mass index (BMI) Z score (kg/m^2^), calculated from the macro created by the World Health Organization, as well as WC (cm). The normal weight group was classified as −2 to 1 standard deviations (SD), the OW group had >1 to 2 SD, and the OB group had >2 SD from the normal value [26].

#### 2.2.2. Cardiometabolic Risk Markers

Lipid, glucose, and insulin concentrations were determined under fasting conditions (at least 12 h) from serum samples. Glucose (mg/dL), total cholesterol, low-density lipoprotein cholesterol (LDL-C, mg/dL), high-density lipoprotein cholesterol (HDL-C, mg/dL), and triglyceride (mg/dL) concentrations were determined by standard methods and measured with the Clinical Chemistry System ILab 300 Plus^®^ equipment. Insulin (μU/mL) concentration was measured using a chemiluminescent method in the IMMULITE^®^ immunoassay analyzer. The homeostasis model assessment of insulin resistance (HOMA-IR) was estimated using the Hosker and Matthews model [27]. All of these variables were considered continuous.

### 2.3. Independent Variables

#### 2.3.1. Fructose Intake from Dietary Information

Expressed as a percentage of the caloric contribution from fructose to total dietary energy, the dietary intakes of children were collected through a short-term Semi-quantitative Food Frequency Questionnaire (SFFQ) involving 107 foods. The SFFQ was designed based on Willet’s methodology [28]. Interviewers asked participants to recall all foods (and portions) consumed in the month prior to the interview. The portion sizes specified by the interviewers were based on the average weight value assigned to each food item for this population. We constructed a specific food-composition database to estimate the energy, macronutrient, and micronutrient intake data collected from the SFFQ. It was based on the Mexican Food Database (BAM, Spanish acronym) with 1721 foods, which was compiled and updated in 2016 by the Center for Nutrition and Health Research of the INSP [29]. To construct our specified database for the SFFQ, we used the following steps: (1) We selected the BAM foods that represented each food item on the SFFQ food list. For example, in the case of ‘mango’, we selected all BAM mango types (*criollo, petacón*, and *manila*). (2) We estimated the energy and nutrient content of the dish items per 100 g of preparation according to their raw ingredients (based on standardized Mexican recipes). Using the constructed food composition database and the weights of the portion sizes (from the SFFQ), we estimated the average daily intakes of energy and nutrients of the children. The quantity of consumed food (in net grams) was determined by considering the density factor for beverages and the edible portion factor for fruits, vegetables, and meats. The daily dietary intake (including fructose and energy) per person was calculated using Stata version 13 software. Finally, the percentage of fructose (from all food and beverage sources) consumed relative to the total energy intake was categorized into tertiles (low, medium, and high).

#### 2.3.2. RA of *L. reuteri*

Genomic DNA was isolated from 200 mg of feces using the QIAamp^®^ DNA Stool (Qiagen, Hilden, North Rhine-Westphalia, Germany) kit. DNA concentrations and purity were determined at an absorbance of 260/280 nm by the Thermo Scientific NanoDrop 1000 Lite spectrophotometer (Wilmington, DE, USA). DNA was stored at −20 °C until use. The RA of *L. reuteri* was determined by qPCR using previously reported primers [30] and universal primers as normalizers of abundance of *L. reuteri* [31]. The PCR mixture of 10 µL contained 1 µL of DNA (5 ng), 5 μL of 2X Maxima SYBR Green/ROX qPCR Master Mix (Thermo Fisher Scientific, Foster City, CA, USA), 1 µL of each primer at 5 pmol/µL, and 2 µL of DNAse/RNAse free water. The temperatures for *L. reuteri* gene amplification were as follows: 10 min at 95 °C, 40 cycles of 15 s at 95 °C, and 1 min at 56 °C. Those for universal primers were as follows: 10 min at 95 °C, 40 cycles of 15 s at 95 °C, and 1 min at 58 °C. Specific gene amplifications and the absence of primer dimers were determined by performing melting curve analyses in all cases. Duplicated sample reactions and a negative control were included in each plate. RA units of *L. reuteri* were obtained by the comparative method 2-ΔCt (*L*. *reuteri* Ct—universal Ct) [32]. This variable was analyzed as continuous and categorical in tertiles.

### 2.4. Covariates

We adjusted the models for the following characteristics: age, sex, first-degree family history of OB (FHO; one or both parents with OB), and leisure time physical activity (LTPA).

#### 2.4.1. Leisure Time Physical Activity (LTPA)

The time spent by children in physical activity during the previous month was evaluated through a questionnaire adapted to this population [33]. Information regarding physical activity performed regularly in the last month was collected. The types and duration were converted to metabolic equivalents (MET/hour/week) by the software “Food Processor Nutrition and Fitness” version 10.12.0 (ESHA Research Inc., Salem, Oregon, OR, USA) [34].

#### 2.4.2. Sociodemographic Information

Trained interviewers collected the socioeconomic, demographic, and personal pathological and hereditary information.

### 2.5. Statistical Analysis

We used χ^2^ tests for frequencies and one-way ANOVAs with Bonferroni post-test correction for continuous variables to assess the significance of differences in normal nutritional status versus OW and OB status according to selected characteristics. Multiple linear models were used to evaluate the direct association between fructose intake and *L. reuteri* RA. Path analysis was used to evaluate direct and indirect associations (to examine if adiposity acts as a mediator) between *L. reuteri* RA and fructose intake with cardiometabolic risk markers (glucose, insulin, HOMA-IR, triglycerides, total cholesterol, HDL-C, and LDL-C). Given the large number of outcome variables (eight) for the path models and covariates, we used principal components and factor analyses to identify variables greatly related to each other. We identified the internal patterns of association among outcome variables, reducing them to three groups in the first phase: group I included BMI and WC; group II included total cholesterol, HDL-C, LDL-C, and triglyceride concentrations; and group III included glucose and insulin concentrations. Finally, we also considered two models based on the conceptual map of the study. The first model incorporated all indicators of adiposity (BMI and WC, based on group I), and the second model incorporated all cardiometabolic risk markers (collapsed groups II and III for these indicators).

The final models included only the variables that influenced the explanations of the models. The goodness of fit of the data was evaluated for all models. All statistical analyses were performed using Stata software: Release 13 (College Station, TX, USA). Statistical significance was defined as a *p*-value < 0.05.

## 3. Results

Eligible children with complete information on adiposity (*n* = 1018) and cardiometabolic risk markers (*n* = 945) who had available data on fructose intake, *L. reuteri* RA, and adjusted covariates (age, sex, FHO, and LTPA) represented 93.65% and 87% of the total sample, respectively. We found that the sample of children included in our analysis had a smaller proportion of females (44.71 vs. 57.28, *p <* 0.0001), higher prevalence of FHO (54.97% vs. 47.73%, *p* = 0.008), and a higher proportion of children with a status of OW/OB (53.08% vs. 45.45%, *p* = 0.02) than the sample of children excluded from the study (Table S1).

Children with OB were mainly male, and a high proportion of these children had FHO (*p* < 0.05). Serum triglyceride, insulin, total cholesterol, and LDL-C concentrations and HOMA-IR were significantly higher, while the HDL-C concentration was significantly lower (*p* < 0.05) in children with OW and OB than in children of normal weight. Likewise, children with OB and OW had higher concentrations of *L. reuteri* RA than children of normal weight. However, children with OB had higher fructose intakes and higher percentage contributions of fructose to the diet than children of normal weight and children with OW (Table 1).

**Figure 2:**
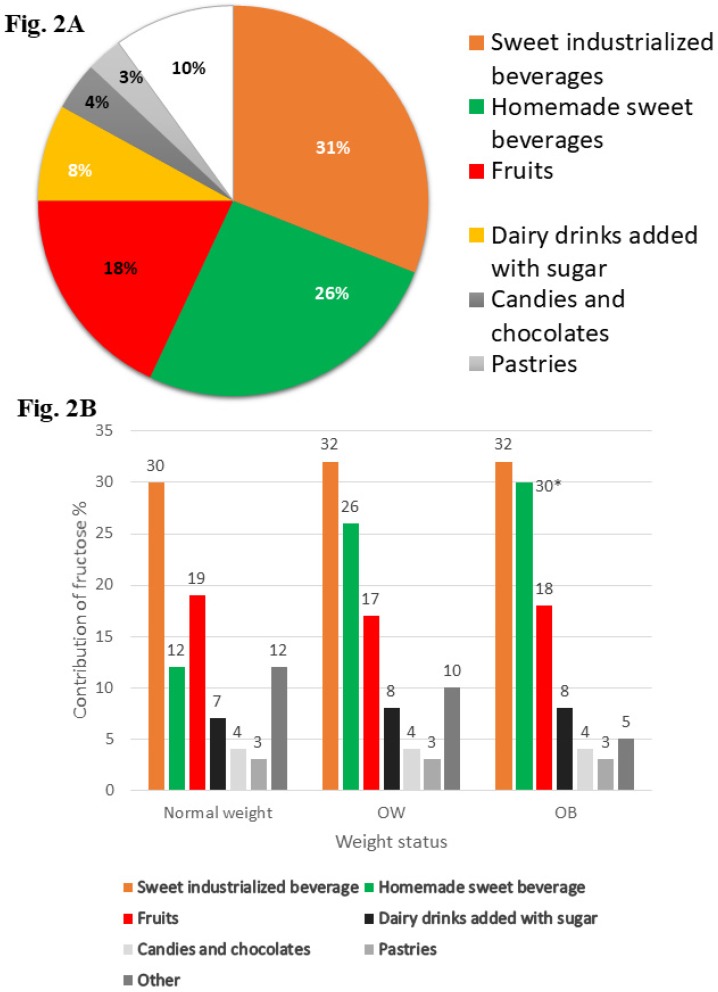
Main food and beverage that provide fructose to diet of children. (**A**) Show the percentage of contribution from main foods and beverage to the diet of school-aged children. (**B**) Show the percentage of contribution from main foods and beverage to the diet of school-aged children by weight status. One-way ANOVA with Bonferroni posttest. Marginally significant difference * *p* < 0.10. OW, overweight; OB, Obesity

Sugar-sweetened beverages (industrialized and homemade) and sweetened lactic beverages were the beverages with the highest percentages of fructose consumed more frequently by children with OW/OB compared with those by children of normal weight (Figure 2). However, there were only marginally significant differences in consumption of homemade sweet beverages among children with obesity and those of normal weight, and there were no statistically significant differences for other beverages.

A high *L. reuteri* RA (*p* < 0.0001) (BMI for age *Z* score and WC) and high fructose contribution to the diet were directly and positively associated with greater adiposity (BMI for age *Z* score *p* < 0.02, and WC *p* < 0.001) (Table 2 and Figure S1A). However, we did not find any direct association between fructose and *L. reuteri* RA.

The associations between a high *L. reuteri* RA and a high percentage of fructose contribution to the diet with elevated serum concentrations of cardiometabolic risk markers (such as glucose, insulin, triglycerides, total cholesterol, LDL-C, and HOMA-IR, *p* < 0.01) and a low serum concentration of HDL-C (*p* < 0.01), were all mediated by adiposity (Table 3 and Figure S1B). The results of the goodness-of-fit test for models are presented in Figure S1A (*p* > chi2 = 0.422, RMSEA = 0.005, GFI = 1.000, SRMR = 0.014) and Figure S1B (*p* > chi2 = 1.000, RMSEA ≤ 0.001, GFI = 1.000, SRMR = 0.004).

## 4. Discussion

We reported that the presence of both high *L. reuteri* RA and high-tertile fructose intake in school-aged children were directly associated with adiposity indicators (BMI and WC) (*p* = 0.009 and *p* < 0.05) and indirectly with most of the cardiometabolic markers when standardized by waist circumference. Fructose has been associated with multiple deleterious effects on health when consumed excessively [35]. Rippe et al. showed an association between 10 weeks of fructose intake and an increase in the intra-abdominal fat volume in adults [36].

In children, there have only been a few studies on fructose consumption through sugary drinks. Ludwig et al. [9] found a 60% higher risk of OB for each additional serving of sweetened beverages consumed per day in American school-aged children (OR, 1.60; 95% CI, 1.14–2.24; *p* = 0.02). In a similar study, an increase in the risk of OW and OB after drinking sugar sweetened beverages was also reported (OR, 2.57; 95% CI, 1.06–3.38; *p* = 0.05) [10].

Recently, different types of foods besides sweetened beverages—known as discretionary foods—are the primary sources of added sugars and fructose in the diet. In the present study, we identified that the major types of discretionary food with a major fructose contribution consumed in this population were industrialized and homemade sweetened beverages and sweetened lactic beverages. Fruit had a low contribution to fructose intake. In spite of the carbohydrates contained in fruits, this group of food does not have the same effect as discretionary food (processed and ultraprocessed), due to the presence of fiber, minerals, antioxidants, vitamins, and phytochemicals [37], which counteract the deleterious effects of excessive fructose. Fructose is associated with the promotion of OB because it is a source of glycerol-3-phosphate and acetyl-CoA not regulated by important metabolic pathways (such as glycolysis, gluconeogenesis, and lipogenesis) [36]. Moreover, fructose may play a role in the development of OB because it is an orexigenic promoter, which leads to increased food intake [35,38]. Furthermore, human studies have documented that excessive consumption of fructose increases uric acid levels, promotes insulin resistance, alters endothelial dysfunction, and produces oxidative stress [39].

OB is associated with specific intestinal microbiota patterns. A high RA of the phylum Firmicutes and a low RA of Bacteroidetes are associated with OB in adults [40] and children [23]. Moreover, a higher concentration of the genus *Lactobacillus* was observed in patients with OB than in controls with anorexia (*p* = 0.03) of those of normal weight (*p* = 0.01) [41]. Particularly, the abundance of certain strains of *L. reuteri* is associated with body weight gain in animals and humans. In piglets and mice, an increase in body weight after the administration of specific strains of L*. reuteri*, such as L6798 and I5007, was noted [42,43,44]. In humans, the association of *L. reuteri* RA with body weight gain has only been evaluated in French adults. Million et al. found that a high *L. reuteri* RA was associated with OB (OR, 1.79; 95% CI, 1.03–3.10; *p* = 0.04) and positively correlated with BMI (*r* = 0.85; 95% CI, 0.12–0.58; *p* = 0.02) [21,22]. However, Fak et al. evaluated three different strains of *L. reuteri* with different effects on mice weight. *L. reuteri* ATCC PTA 4659 was associated with less weight gain, the 236 strain had an anti-OB effect, and *L. reuteri* L6798 was associated with high weight gain [44,45]. Regarding strain 263, Chen et al. reported an anti-OB effect of *L. reuteri* in rats fed a high-energy diet [46].

In the present study, we found an association between *L. reuteri* RA and OB in children. A probable explanation for this relation is the heterofermentative lactic acid capacity of some *L*. *reuteri* strains to grow in different types of sugars and to degrade nondigestible carbohydrates, generating short chain fatty acids (SCFA) like acetate, butyrate, and propionate. Although SCFAs are neither purely obesogenic nor antiobesogenic, acetate, for example, has the highest absorption in the colon and is transported to the liver, contributing to lipogenesis [17,47,48]. This mechanism is plausible; however, experimental studies have shown an opposite effect in organisms [49]. In contrast, these results on *L. reuteri* should be carefully examined because some strains of *L. reuteri* are known to prevent gastrointestinal disorders by inhibiting the growth of various pathogenic microorganisms such as *Escherichia coli*, *Staphylococcus aureus*, *Salmonella typhimurium*, *Helicobacter pylori*, and rotavirus [50].

Strains such as *L. reuteri* DSM17938 have been associated with the reduction of multi-organ inflammation and intestinal microbiota remodeling in mice [45]. Meanwhile, in humans undergoing insulin therapy for diabetes, this strain did not show effects on HbA1c [51], and *L. reuteri* NCIMB 30242 has been shown to have hypocholesterolemic effects [52,53]. In addition, in healthy volunteers, the *L. reuteri* SD5865 increased the secretion of insulin, C peptide, and intestinal peptides, without modifying glucose tolerance and insulin sensitivity [54]. More research on *L. reuteri* is needed to improve knowledge on its effect on health.

The proposed associations are biologically plausible because the production of pro-inflammatory cytokines and activation of the immune response are generated in hyperplasic and hypertrophic adipose tissue following the excessive accumulation of triglycerides. Both cytokine and immune response activation have been implicated in oxidative stress, and insulin resistance is associated with dyslipidemia [55,56].

Two studies on healthy adults reported that a high fructose intake increases the serum triglyceride concentration in the fasting and postprandial periods without any body weight or BMI changes [57,58]. On the contrary, fructose restriction for 9 days significantly decreased serum triglyceride, LDL-C, glucose, and insulin concentrations in Hispanic or African American adolescents and children. The authors concluded that the low concentration of fructose used may be the cause of the decrease in weight in patients after completion of the study [59].

We discarded the possibility of a potential selection bias despite including only 73.3% of the total study population, because we did not find differences between groups after comparing the mean contribution of fructose between children excluded and included from the study (4.18% vs. 4.19%, *p =* 0.87). (Table S1). A potential measurement error in the fructose intake may be due to some details not being obtained from diet in the SFFQ, and because this method is inadequate for quantifying consumption; however, SFFQ was used in the same way for all children (normal, OW, and OB), avoiding a differential measurement error. Despite the inherent underestimation associated with the SFFQ, the method allows the different categories of nutrient intake to be compared.

Another consideration is that we did not find any significant differences in energy intake between overweight or obese children in comparison with normal weight children. Under-reporting of energy intake has been previously documented both in overweight and obese adults as well as in children. A potential explication for this is due to the health image of specific food items; those with a negative health image are more likely to be under-reported in comparison to foods with a positive health image, such as vegetables, which tend to be reported in excess. However, the energy intake estimates in this study as well as those used in the National Health and Nutrition Survey 2012 (ENSANUT-2012, Spanish acronym) were obtained through SFFQ, and these both had a low prevalence of under-reporting (12.4% and 14%, respectively). On the other hand, the SFFQ measures the current diet (at last month), and given the overwhelming obesogenic food environment, this condition may affect the variation in consumption among normal weight versus overweight or obese children. Likewise, despite the small differences found between the percentages of energy contribution from fructose in foods, these differences are relevant if we consider that exposure at these levels is continuous (and for a long time). We found a difference in the energy contribution of fructose from industrialized sweetened beverages among normal weight versus obese children of 2% or more, and for sugary homemade beverages, the energy contribution from fructose ranged from 12% for normal weight children to 30% for those with obesity. In contrast, normal weight children had a higher fruit intake compared to obese children. On the other hand, we speculate that these values may be underestimated by the under-reporting of unhealthy foods in overweight or obese children in comparison with normal weight children. Therefore, the estimated differences in the percentages of energy contribution of fructose for some foods may be less than the real differences, and due to the obesogenic food environment, there may be a lower variability in food intake.

Also, another potential limitation of the SFFQ that must be considered is the effect of surrogate information for children aged 6 to 11 years. In this respect, the questionnaire was applied to the caregiver in the presence of the child so that the child could have the opportunity to add information. The above was done in order to decrease under-reporting [60,61].

## 5. Conclusions

Our results suggest a potential association between a high *L. reuteri* RA and fructose intake with greater adiposity and metabolic alterations in children. However, more research in this area is required to clarify and understand which *L*. *reuteri* strains may pose health risks for children. These findings contribute to the documentation of potential risk factors that may be associated with increased OB and metabolic alterations in school-aged children.

## Figures and Tables

**Table 1:** General characteristics by BMI status of children from Mexico City ^1^.

Characteristics ^2^	BMI Status		
	Normal Weight (*n* = 510)	OW (*n* = 287)	OB (*n* = 290)
Age (year)	9.19 ± 1.76 a	9.70 ± 1.72 b	9.60 ± 1.80 b
Girls (%)	48.43 a	46.70 a	36.21 b
LTFA (MET)	441.81 ± 376.77 a	444 ± 411.80 a	448.34 ± 409.40 a
FHO (%)	47.74 a	57.50 b	65.20 c
BMI for age *Z*-score	−0.11 ± 0.76 a	1.60 ± 0.60 b	2.70 ± 0.50 c
WC	57.45 ± 5.55 a	68.75 ± 7.60 b	78.80 ± 9.15 c
Glucose (mg/dL)	81.45 ± 9.80 a	81.92 ± 8.60 a	83.70 ± 8.90 b
Triglycerides (mg/dL)	73.30 ± 29.07 a	101.70 ± 48.60 b	118.70 ± 53.40 c
Total cholesterol (mg/dL)	155.56 ± 32.18 a	162.03 ± 33.20 b	162 ± 33.30 b
HDL-C (mg/dL)	54.80 ± 11.99 a	50.80 ± 12.77 b	45.5 ± 11.5 c
LDL-C (mg/dL)	98.06 ± 24.25 a	107.50 ± 26.40 b	109.80 ± 27.40 b
Insulin (μU/mL)	4.83 ± 3.63 a	8.60 ± 7.10 b	11.30 ± 10.40 c
HOMA-IR	0.97 ± 0.76 a	1.77 ± 1.50 b	2.40 ± 2.35 c
**Exposure variables**			
Energy intake (kcal)	2158.48 ± 722.13 a	2205.40 ± 766.56 a	2226.80 ± 824.90 a
Fructose intake (g)	24.31 ± 11.98 a	25.80 ± 13.50 a	27.50 ± 17.50 b
Fructose contribution (%)	4.05 ± 1.41 a	4.23 ± 1.60 a	4.40 ± 1.70 b
***L. reuteri* (RA)**	**0.007 ± 0.025 a**	**0.17 ± 1.4 b**	**0.40 ± 1.74 c**

^1^ Original to this manuscript. ^2^ Values are means ± SD or percentages. BMI for age *Z*-score, *Z*-score body mass index by age; WC, waist circumference; LTFA, leisure time physical activity; MET, Metabolic Equivalent of Task; FHO, family history of obesity; HDL-C, high density lipoprotein; LDL-C, low density lipoprotein; HOMA-IR, homeostasis model assessment of insulin resistance; OW, overweight; OB, Obesity; RA, relative abundance; WC, waist circumference. One-way ANOVA with Bonferroni posttest or Chi square for continuous or categorical variables, respectively. Different superscript letters (a, b, c) differ significantly at *p* < 0.05. In bold statistically significant differences.

**Table 2:** Direct and indirect effects of *L. reuteri* RA and diet fructose contribution on adiposity indicators and cardiometabolic markers in children of Mexico City ^1^.

	Diet Fructose Contribution (%)						Relative Abundance of *L. reuteri*					
	Medium-Tertile ^4^ (3.96 ± 0.33)			High-Tertile ^4^ (5.85 ± 1.40)			Medium-Tertile ^5^ (0.0007 ± 0.0004)			High-Tertile ^5^ (0.50 ± 1.86)		
	Path Coefficient	95% CI	*P* Value	Path Coefficient	95% CI	*P* Value	Path Coefficient	95% CI	*P* Value	Path Coefficient	95% CI	*P* Value
**Adiposity Indicators**												
**Direct Effect**												
BMI for age *Z*-score	−0.07 ^2^	−0.30, 0.12	0.50	0.24 ^2^	0.04, 0.44	0.02	0.27 ^2,6^	0.07, 0.47	0.009	0.52 ^2,6^	0.32, 0.72	<0.001
WC, cm	0.30 ^2^	−1.2, 1.75	0.70	2.40 ^2^	0.95, 3.84	0.001	1.60 ^2,6^	0.12, 3.04	0.03	3.40 ^2,6^	1.95, 4.90	<0.001
**Indirect Effects (Via RA *L reuteri*)**												
BMI for age *Z*-score	0.02 ^2^	−0.12, 0.05	0.25	0.01 ^2^	−0.02, 0.04	0.51	-	-		-	-	-
WC, cm	0.11 ^2^	−0.08, 0.31	0.25	0.06 ^2^	−0.12, 0.25	0.60	-	-		-	-	-
**Total Effects**												
BMI for age *Z*-score	−0.05 ^2^	−0.25, 0.15	0.60	0.24 ^2^	0.05, 0.45	0.02	0.27 ^2,6^	0.07, 0.47	0.009	0.52 ^2,6^	0.32, 0.72	<0.001
WC, cm	0.40 ^2^	−1.06, 1.88	0.54	2.45 ^2^	1.00, 3.90	0.001	1.60 ^2,6^	0.12, 3.04	0.03	3.40 ^2,6^	1.95, 4.90	<0.001

^1^ BMI for age *Z*-score, body mass index for age Z score; Ref, reference; WC, waist circumference. ^2^ Adjusted for age, sex, family history of obesity, and leisure time physical activity (*n* = 1018). ^4^ Reference was low-tertile of contribution percentage of fructose to diet intake (2.75 ± 0.50). ^5^ Reference was low-tertile of relative abundance of *L. reuteri* (0.00006 ± 0.00005). ^6^ Total effects correspond to direct effects, because no significant indirect effects were found in these models. In bold statistically significant differences.

**Table 3:** Direct and indirect effects of *L. reuteri* RA and diet fructose contribution on cardiometabolic markers in children of Mexico City ^1^.

	Diet Fructose Contribution (%) ^2^						Relative Abundance of *L. Reuteri* ^2^					
	Medium-Tertile ^3^			High-Tertile ^3^			Medium-Tertile ^4^			High-Tertile ^4^		
	Path Coefficient	95% CI	*P* Value	Path Coefficient	95% CI	*P* Value	Path Coefficient	95% CI	*P* Value	Path Coefficient	95% CI	*P* Value
**Cardiometabolic Markers**												
**Direct Effect**												
Glucose, mg/dL	1.24	−0.17, 2.65	0.08	0.34	−1.06, 1.75	0.63	−0.27	−1.70, 1.14	0.70	0.23	−1.18, 1.65	0.75
Insulin, μU/mL	0.49	−0.51, 1.50	0.96	0.87	−0.13, 1.88	0.09	−1.00	−2.01, 0.11	0.06	0.007	−1.01, 1.02	0.98
HOMA-IR	0.13	−0.09, 0.36	0.25	0.20	−0.03, 0.43	0.09	−0.22	−0.45, 0.01	0.06	0.03	−0.20, 0.26	0.80
LDL-C, mg/dL	2.70	−1.33, 6.73	0.18	−1.27	−5.30, 2.76	0.53	−0.32	−4.37. 3.71	0.87	−0.70	−4.75, 3.35	0.73
HDL-C, mg/dL	0.03	−1.76, 1.81	0.97	0.50	−1.28, 2.30	0.60	−0.45	−2.24, 1.34	0.62	0.19	−1.60, 2.00	0.83
Triglycerides, mg/dL	1.97	−4.31, 8.27	0.53	−2.60	−8.88, 3.69	0.41	−2.47	−8.79, 3.83	0.44	−6.03	−12.36, 0.30	0.06
**Indirect Effect (Via Waist Circumference)**												
Glucose, mg/dL	−0.03	−0.20, 0.12	0.70	0.14	−0.03, 0.33	0.11	0.12	−0.03, 0.27	0.10	0.25	0.02, 0.50	0.03
Insulin, μU/mL	0.06	−0.42, 0.55	0.80	**0.75**	**0.25, 1.24**	**<0.01**	**0.44**	**−0.04, 0.93**	**0.07**	**0.91**	**0.41, 1.41**	**<0.001**
HOMA-IR	0.01	−0.09, 0.11	0.78	**0.15**	**0.05, 0.26**	**<0.01**	**0.09**	**−0.01, 0.20**	**0.08**	**0.19**	**0.08, 0.30**	**<0.001**
LDL-C, mg/dL	−0.24	−1.09, 0.61	0.60	**1.02**	**0.12, 1.93**	**0.03**	**0.90**	**0.04, 1.75**	**0.04**	**1.80**	**0.80, 2.80**	**<0.001**
HDL-C, mg/dL	−0.12	−0.80, 0.54	0.71	**−1.03**	**−1.71, −0.35**	**<0.01**	**−0.60**	**−1.26, 0.08**	**0.08**	**−1.21**	**−1.91, −0.50**	**0.001**
Triglycerides, mg/dL	−0.17	−3.37, 3.02	0.91	**4.62**	**1.40, 7.84**	**<0.01**	**3.24**	**0.03, 6.45**	**0.05**	**6.58**	**3.30, 9.90**	**<0.001**
**Total Effect**												
Glucose, mg/dL	1.21	−0.20, 2.64	0.09	0.50	−0.92, 1.90	0.50	−0.14	−1.60, 1.30	0.83	0.48	−0.92, 1.90	0.50
Insulin, μU/mL	0.56	−0.55, 1.68	0.32	**1.62**	**0.50, 2.73**	**<0.01**	−0.55	−1.68, 0.56	0.33	0.92	−0.19, 2.04	0.10
HOMA-IR	0.14	−0.10, 0.40	0.25	**0.35**	**0.10, 0.61**	**<0.01**	−0.12	−0.38, 0.12	0.31	0.22	−0.02, 0.50	0.09
LDL-C, mg/dL	2.46	−1.64, 6.56	0.24	−0.25	−4.33, 3.85	0.90	0.60	−3.55, 4.68	0.80	1.10	−3.00, 5.20	0.60
HDL-C, mg/dL	−0.10	−2.00, 1.80	0.92	−0.54	−2.44, 1.35	0.57	−1.04	−2.95, 0.87	0.30	−1.02	−2.92, 0.88	0.30
Triglycerides, mg/dL	1.81	−5.22, 8.84	0.60	2.02	−4.98, 9.03	0.60	0.76	−6.3, 7.81	0.83	0.55	−6.47, 7.57	0.94

^1^ Original to this manuscript. HOMA-IR, homeostasis model assessment of insulin resistance; LDL-C, low density lipoprotein; HDL-C, high density lipoprotein. ^2^ Adjusted for age, sex, family history of obesity and leisure time physical activity (*n* = 945). ^3^ Reference was low-tertile of contribution percentage of fructose to diet intake. ^4^ Reference was low-tertile of relative abundance of *L. reuteri*. In bold statistically significant differences.

## Supplementary Materials

The following are available online at https://www.mdpi.com/2072-6643/11/6/1207/s1, Figure S1: Path model of direct and indirect association between fructose intake tertiles and relative abundance of *L. reuteri* tertiles with adiposity and cardiometabolic markers in the cohort Mexican children. Table S1: Comparison of general characteristics of main analysis sample with information of the RA *L. reuteri* and Fructose intake and individuals without information.

## Abbreviations

BMI	Body Mass Index
HDL-C	High Density Lipoprotein
FHO	Family History of Obesity
HOMA-IR	Homeostasis Model Assessment of insulin resistance
LDL-C	Low Density Lipoprotein
LTPA	Leisure Time Physical Activity
METs	Metabolic Equivalent of Task
OB	Obesity
OW	Overweight
RA	Relative Abundance
SFFQ	Semi-Quantitative Food Frequency Questionnaire
WC	Waist Circumference
