# Supplementary material for: High Relative Abundance of Lactobacillus reuteri and Fructose Intake are Associated with Adiposity and Cardiometabolic Risk Factors in Children from Mexico City
Source: Nutrients. 2019 May 28;11(6):1207. doi: 10.3390/nu11061207 (PMC6627236; doi:10.3390/nu11061207)
Supplement: Supplementary file 1 [file nutrients-11-01207-s001.zip › SUPPLEMENTARY/3_Supplementary TABLE 1.docx]

**SUPPLEMENTARY TABLE 1. Comparison of general characteristics of main analysis sample with information of the RA *L. reuteri* and Fructose intake and individuals without information^1^**

| Variables | Main analysis sample  (n=1,087) |  | Sample losses (n= 309) |  | *P* value^*^ |
| --- | --- | --- | --- | --- | --- |
| Age, *y* | 9.43±1.77 |  | 9.62±1.81 |  | 0.08 |
| Girls, % | 44.71 |  | 57.28 |  | <0.0001 |
| Leisure time physical activity, MET | 444.13±394.80 |  | 428.38±400.36 |  | 0.55 |
| Family history of obesity, % | 54.97 |  | 47.73 |  | 0.02 |
| Fructose contribution, % | 4.18±1.55 |  | 4.19±1.55 |  | 0.87 |
| Overweight, % | 26.40 |  | 20.70 |  | 0.05 |
| Obese, % | 26.68 |  | 24.75 |  | 0.06 |

^1^ Original to this manuscript.

^2^ Values are means ± SD or percentages; MET, Metabolic Equivalent of Task; RA, relative abundance. * Student’s *t* test or Chi square for continuous or categorical variables, respectively, *p-value*
