# Supplementary material for: High Relative Abundance of Lactobacillus reuteri and Fructose Intake are Associated with Adiposity and Cardiometabolic Risk Factors in Children from Mexico City
Source: Nutrients. 2019 May 28;11(6):1207. doi: 10.3390/nu11061207 (PMC6627236; doi:10.3390/nu11061207)
Supplement: Supplementary file 1 [file nutrients-11-01207-s001.zip › SUPPLEMENTARY/4_Supplemental Figure 1.pdf]

Supplemental Fig. 1 A

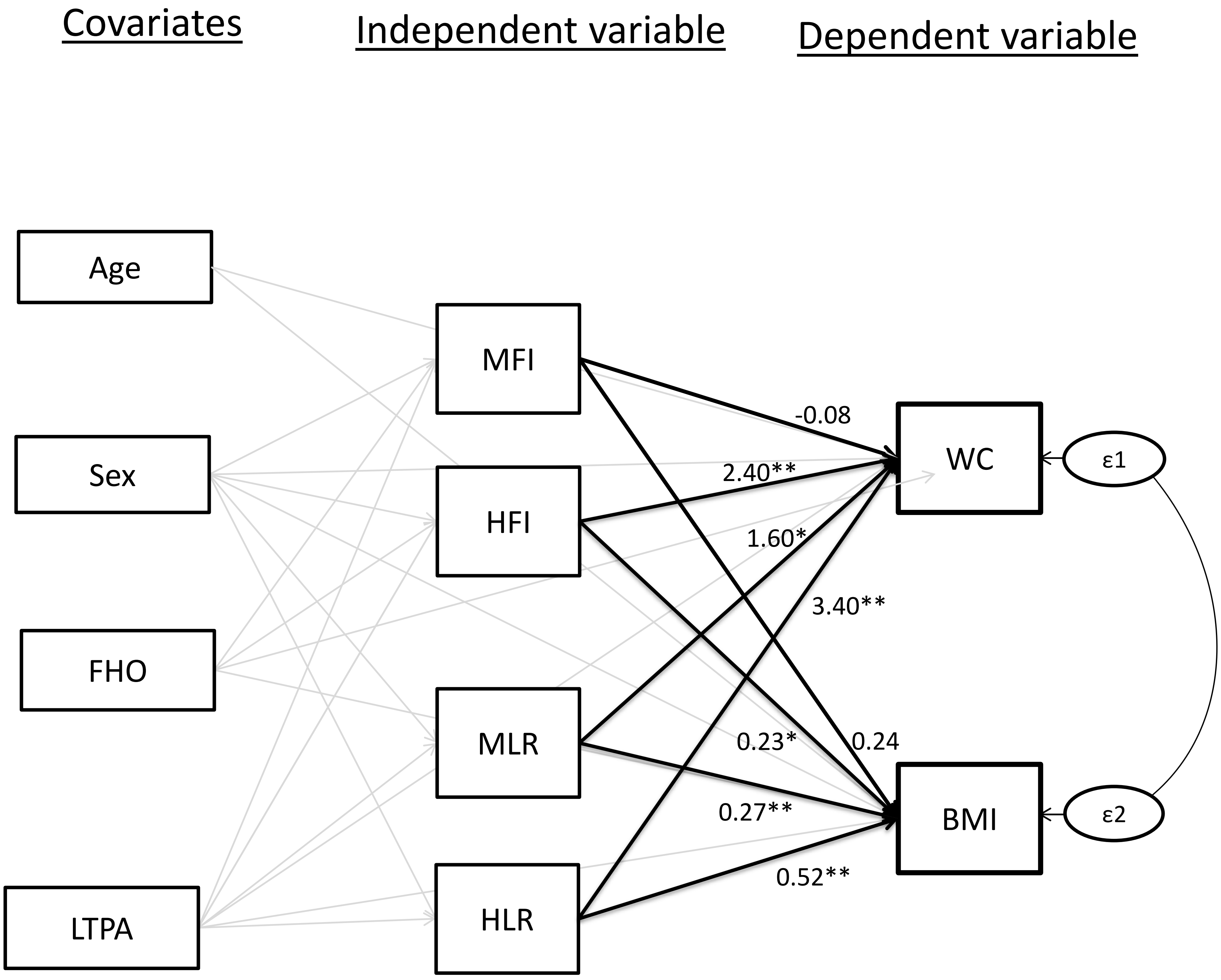

Supplemental Fig. 1 B

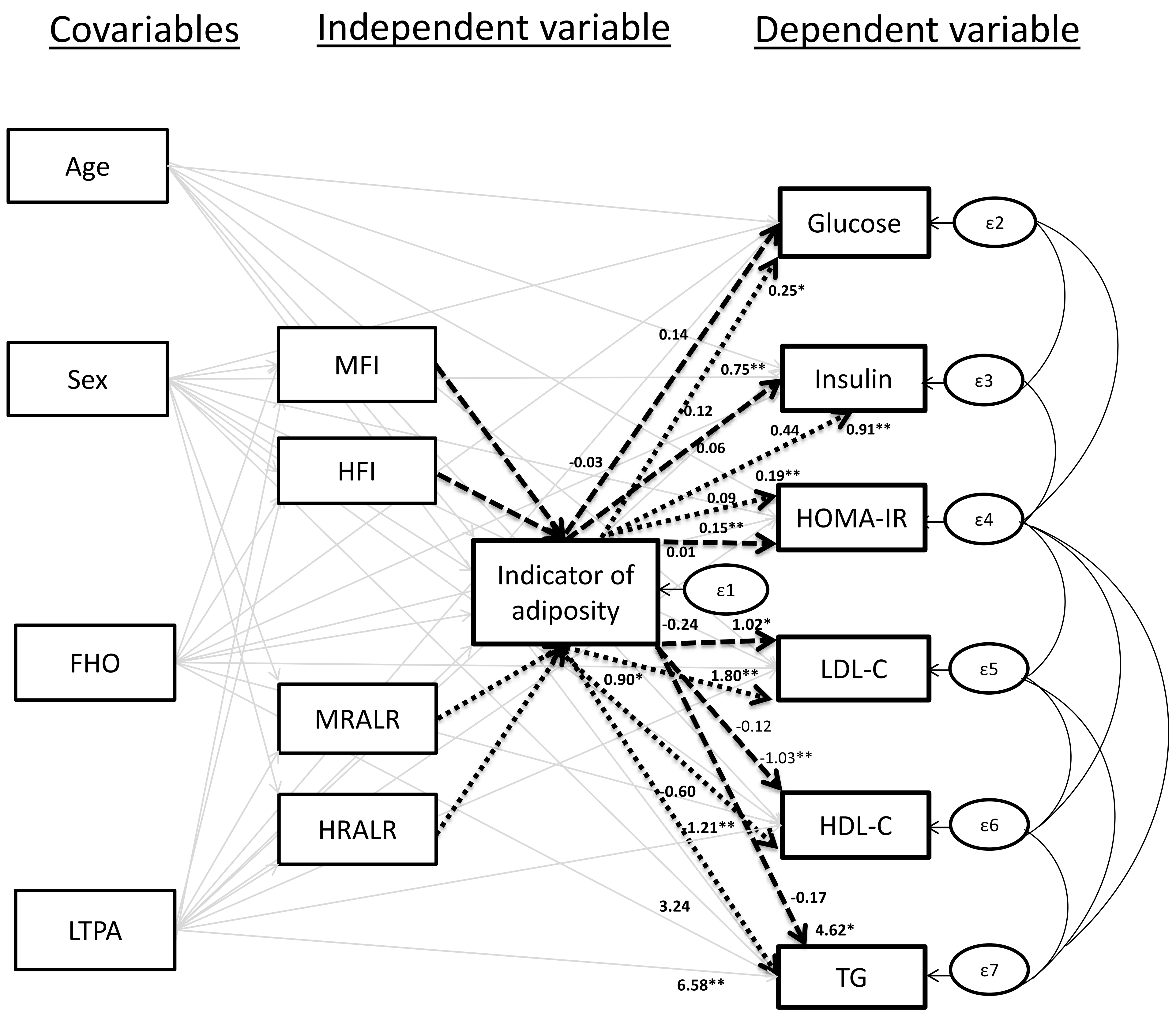

Path model of direct and indirect association between fructose intake tertiles and relative abundance of *L. reuteri tertiles* with adiposity and cardiometabolic markers in the cohort Mexican children.

Arrows represent pathways among variables that were statistically significant. The bold arrows and the path coefficients show the significant associations which are relevant to the study objectives (*p <0.05; **p < 0.01). LFI and LLR were the reference. **A** highlights the statistical significant direct relation between fructose intake tertiles and relative abundance of *L. reuteri* (in tertiles) with adiposity. **B** highlights the indirect relation between fructose intake tertiles and relative abundance of *L. reuteri* (in tertiles) with glucose (mg/dL), insulin (µU/mL), HOMA-IR, LDL-C (mg/dl), HDL-C (mg/dL) and triglycerides (mg/dL) which were mediated by adiposity (WC).

FHO, family history of obesity; HDL-C, high density lipoprotein; HFI, High fructose intake; HLR, high relative abundance of *L. reuteri*; HOMA-IR, homeostasis model assessment of insulin resistance; LTPA, Leisure time physical activity; LDL-C, low density lipoprotein; LFI, low fructose intake; LLR, low relative abundance of *L. reuteri*; MFI, medium fructose intake; MLR, medium relative abundance of *L. reuteri*; TG, Triglycerides
